# Deep-learning-based analysis of preoperative MRI predicts microvascular invasion and outcome in hepatocellular carcinoma

**DOI:** 10.1186/s12957-022-02645-8

**Published:** 2022-06-08

**Authors:** Bao-Ye Sun, Pei-Yi Gu, Ruo-Yu Guan, Cheng Zhou, Jian-Wei Lu, Zhang-Fu Yang, Chao Pan, Pei-Yun Zhou, Ya-Ping Zhu, Jia-Rui Li, Zhu-Tao Wang, Shan-Shan Gao, Wei Gan, Yong Yi, Ye Luo, Shuang-Jian Qiu

**Affiliations:** 1 Department of Liver Surgery, Liver Cancer Institute, Zhongshan Hospital, and Key Laboratory of Carcinogenesis and Cancer Invasion (Ministry of Education), Fudan University, Shanghai, People’s Republic of China; 2 School of Software Engineering, Tongji University, Shanghai, People’s Republic of China; 3 Department of Radiology, Zhongshan Hospital, Fudan University, Shanghai Institute of Medical Imaging, Shanghai, 200032 People’s Republic of China; 4 Department of General Surgery, Zhongshan Hospital, Fudan University, Shanghai, People’s Republic of China

**Keywords:** Deep learning, Microvascular invasion, Clinical outcome, HCC

## Abstract

**Background:**

Preoperative prediction of microvascular invasion (MVI) is critical for treatment strategy making in patients with hepatocellular carcinoma (HCC). We aimed to develop a deep learning (DL) model based on preoperative dynamic contrast-enhanced magnetic resonance imaging (DCE-MRI) to predict the MVI status and clinical outcomes in patients with HCC.

**Methods:**

We retrospectively included a total of 321 HCC patients with pathologically confirmed MVI status. Preoperative DCE-MRI of these patients were collected, annotated, and further analyzed by DL in this study. A predictive model for MVI integrating DL-predicted MVI status (DL-MVI) and clinical parameters was constructed with multivariate logistic regression.

**Results:**

Of 321 HCC patients, 136 patients were pathologically MVI absent and 185 patients were MVI present. Recurrence-free survival (RFS) and overall survival (OS) were significantly different between the DL-predicted MVI-absent and MVI-present. Among all clinical variables, only DL-predicted MVI status and a-fetoprotein (AFP) were independently associated with MVI: DL-MVI (odds ratio [OR] = 35.738; 95% confidence interval [CI] 14.027–91.056; *p* < 0.001), AFP (OR = 4.634, 95% CI 2.576–8.336; *p* < 0.001). To predict the presence of MVI, DL-MVI combined with AFP achieved an area under the curve (AUC) of 0.824.

**Conclusions:**

Our predictive model combining DL-MVI and AFP achieved good performance for predicting MVI and clinical outcomes in patients with HCC.

**Supplementary Information:**

The online version contains supplementary material available at 10.1186/s12957-022-02645-8.

## Introduction

Hepatocellular carcinoma (HCC) ranks the sixth most common malignancies worldwide and its incidence is increasing annually [1]. Surgical resection, liver transplantation, and locoregional therapies may be potentially curative for HCC patients, whereas post-operative recurrence and metastasis rate remains high, mainly due to the presence of vascular invasion [2, 3]. Recurrence and metastasis are the main reasons for poor prognosis in post-operative HCC patients. Approximately 70% of HCC patients treated with surgical resection develop a recurrence within 5 years [4]. Early recurrences, within 2 years after tumor resection, are frequently attributed to residual intrahepatic metastases.

Microvascular invasion (MVI) is defined as microscopic invasion of tumor cells within a vascular space lined by endothelium like smaller intrahepatic vessels, including micro-vessels of portal vein or hepatic artery and small lymphatic vessels [5]. MVI is among the most vital prognostic factors for HCC and is a major risk indicator for early recurrence during the first 2 years after surgical resection [6–8]. MVI is frequently present in HCC and highly associates with several adverse biological markers, such as high-grade, large tumor size, and elevated serum AFP [9]. The presence of MVI more accurately predicted higher recurrence risk and poor clinical outcomes than factors included in the Milan criteria [6]. A nomogram containing MVI, macrovascular invasion (MaVI), and CA19-9 demonstrated favorable performance in predicting very early recurrence (recurrence within 6 months after surgery) in combined hepatocellular-cholangiocarcinoma after hepatic resection [10]. Moreover, MVI determines the risk for intrahepatic or distant dissemination of malignant cells, and MVI-positive HCCs should potentially be treated with a wider resection margin to eradicate micro-metastases compared with MVI-negative tumors [11, 12]. Surgical resection, combined with adjuvant intervention therapy or targeted therapy, were reported to prolong survival of HCC patients with MVI compared with surgical resection alone [13, 14].

Although macrovascular invasion can be detected with diagnostic imaging, MVI is a histologic finding that can rarely be determined preoperatively [15]. Currently, preoperative prediction of MVI remains challenging, despite several studies claimed that imaging features extracted from computed tomography (CT) and magnetic resonance imaging (MRI) [16–18], as well as serum metabolites [19] were predictive of MVI. Gd-EOB-DTPA-enhanced MRI has been widely used in clinical liver neoplasia examinations, due to its high sensitivity and accuracy in detecting small HCC lesions with a diameter ≤ 2.0 cm [20]. Gd-EOB-DTPA-enhanced MRI was reported to have a high value in predicting presence of MVI in HCC [21]. MR imaging features, including arterial peritumoral enhancement, tumor margins, tumor size were independently associated with MVI [16, 22], while these imaging features were extracted visually by experienced radiologists, limiting its clinical use. Additionally, it was reported that a radio-genomic venous invasion (RVI) predictor, combining imaging features with gene expression, achieved high accuracy in predicting MVI in HCC [17]. Through radiomic analysis of contrast-enhanced CT, Xun Xu et al. developed a computational approach integrating large scale clinic-radiologic and radiomic features to predict MVI and long-term clinical outcomes of patients with HCC [23]. However, these criteria for a preoperative imaging diagnosis of MVI in HCC have not yet been widely recognized. The main reason is that the radiomics method relies heavily on manually annotated precise margins of tumor by experienced radiologists, causing much manpower and time. Besides, multi-parametric MRI has now become an essential part in clinical practice due to its advantages over contrast-enhanced CT in assessing liver neoplasia.

In recent years, with the continuous advancements achieved in computer science, deep learning (DL), with artificial intelligence as its core, has been paid more and more attention in the medical field. Compared with traditional empirical medicine, medical intelligence can integrate a large scale of existing data and experience to facilitate medical diagnosis and treatment. Image recognition is a now mature field in deep learning and has gone into the analysis of medical images, such as the discrimination between benign occupancy and malignant nodules, the location of organs and lesions, and the division of organs and its substructures [24]. Deep learning analysis of H-E scan slices (convolutional neural network, Resnet18) achieved an area under the curve (AUC) of 0.81–0.84 in predicting gastrointestinal tumor microsatellite instability (MSI) [25]. Deep learning also outperformed many experienced dermatologists in melanoma image classification [26–30]. Deep learning neural networks based on magnetic resonance imaging (MRI), X-ray computer tomography (CT), and PET/CT have had great achievements in the characterization of prostate cancer, pulmonary nodules, hepatocellular carcinoma, or benign occupancy [31–38].

Moreover, the accuracy could be further enhanced by the ability of deep learning to quickly compute high-dimensional data, based on real-time disease location and subsequent analysis of dynamic video such as endoscopy [39–42]. Deep learning, combined with molecular expression information, high-throughput sequencing, and multi-group data is also a research area of concern in this field [43, 44].

However, to the best of our knowledge, there have been few attempts to evaluate the diagnostic performance of deep learning in mining MR imaging features for predicting MVI of HCC and long-term clinical outcomes. This study aimed to investigate whether deep learning analysis of preoperative MR imaging could be used to predict MVI, to determine its diagnostic performance and to evaluate whether it is associated with outcome in HCC patients.

## Material and methods

### Study design and patient population

This retrospective study was approved by Zhongshan Hospital Ethics committee and written informed consent was obtained from all patients. All procedures involving human participants were performed in accordance with the 1975 Helsinki Declaration and its later amendments.

We queried our institution’s medical records to derive data from patients who underwent hepatic resection for HCC in year 2015 and year 2018 respectively. The key inclusion criteria for our study were as follows: (1) resectable HCC lesion without macroscopic vascular invasion; (2) underwent preoperative gadoxetic acid–enhanced and DW liver MR imaging within 1 month before surgery; (3) without a history of prior intervention therapy or partial hepatectomy; (4) pathological confirmation of HCC after surgical resection (5) MR imaging quality adequate for analysis. Exclusion criteria included (1) received anti-tumor therapies such as intervention therapy or partial hepatectomy before surgery; (2) incomplete clinical or pathological information.

A total of 321 confirmed cases of HCC were identified, with 149 HCC patients forming the 2015 cohort and 172 patients forming 2018 cohort, according to the inclusion and exclusion criteria. Data of some preoperative laboratory examinations were collected, including liver function tests, hepatitis B and C immunology, serum a-fetoprotein (AFP) level, serum alanine aminotransferase (ALT), aspartate aminotransferase (AST), γ-glutamyl transpeptidase (GGT), serum total bilirubin (TB), conjugated bilirubin (CB), serum albumin (ALB), platelet count (PLT), prothrombin time (PT), and international normalized ratio (INR). The diagnosis of HCC was histologically or clinically confirmed based on the criteria of the American Association for the Study of Liver Diseases (AASLD) [45].

### MR imaging acquisition

All HCC patients underwent preoperative Gadoxetic acid-enhanced MR imaging examination by a 1.5T scanner (Siemens Healthcare, Erlangen, Germany). Image acquisition procedures were performed as previously reported [46]. Namely, six routine MRI sequences included axial T2-weighted imaging(T2WI) with fat suppression, diffusion-weighted imaging (DWI), dynamic three-dimensional T1-weighted volumetric-interpolated breath-hold examination (VIBE) at pre-contrast phase (T1), arterial phase (20–30 s, T1A) , portal venous phase (about 80 s, T1V), delayed phase (3 min, T1D) after injection of 0.025 mmol/kg of gadoxetic acid (Primovist, Bayer Schering Pharma, Berlin, Germany) into the cubital vein, followed by a 20-mL saline flush. Details of MRI acquisition parameters were listed in the Supplementary Table 1.

### Deep learning network architecture and workflow

**Fig. 1:**
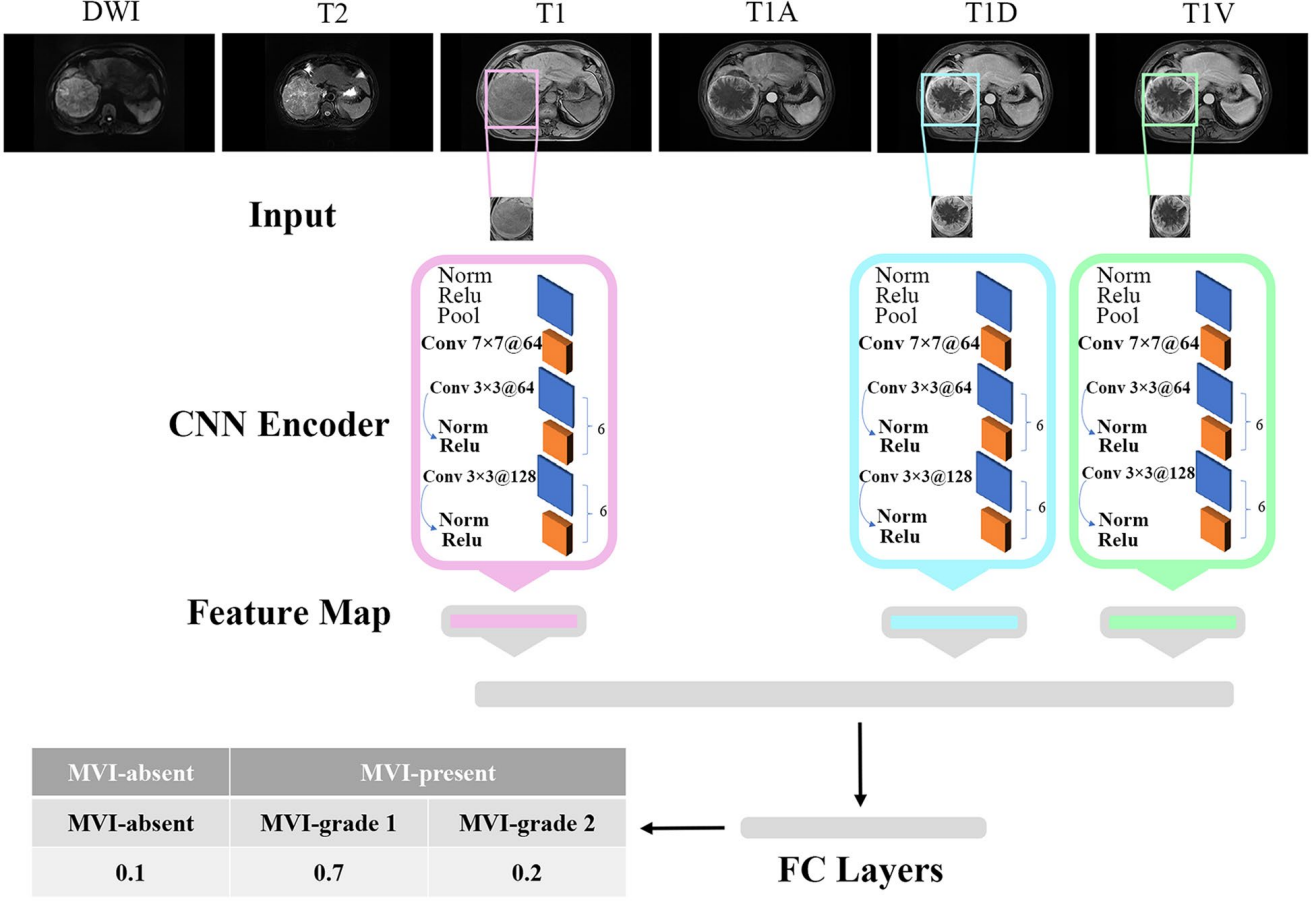
Flowchart of DL model architecture. The DL model has three inputs, which are regions of interest (ROIs) cropped from three raw MRI sequences (T1, T1D, and T1V). In order to make all the ROIs have the same size, we resized them into 320 × 320 pixels, then the processed ROI were input into the conventional neural network (CNN). The separate CNNs were utilized for feature extraction from each of the three ROIs. The features extracted from these three branches were fused and subsequently fed into fully connected (FC) layers combined with a SoftMax classifier to obtain the predicted results. The DL model has three outputs, including predicted results of MVI absent, MVI-grade 1, and MVI-grade 2. MVI-grade 1 and 2 categories were together deemed as MVI present. MVI, microvascular invasion; DL, deep learning; T1, T1-weighted imaging; DWI, diffusion-weighted imaging; T2, T2-weighted imaging; T1A, T1-weighted imaging at arterial phase; T1V, T1-weighted imaging at portal venous phase; T1D, T1-weighted imaging at delayed phase

Considering that different sequences of MRI contain different features to characterize MVI, analyzing the effect of different MR sequences and combinations of pulse sequences for MVI prediction is necessary and important. To analyze MR images from different pulse sequences, we first performed the feature extraction individually and then fused the extracted features to predict the status of MVI. Specifically, the whole procedure was divided into three steps: single MR sequence feature extraction, feature fusion, and feature normalization (Fig. 1).

#### Step 1: feature extraction from single MR sequence

Given a 2D slice image from a single MR sequence, a region of interest (ROI) in a rectangle shape from the original image was cropped by two experienced doctors. In order to focus on the boundary region of the tumor, we enlarged the ROI by 5–10 pixels at every boundary of the rectangular ROI. In other words, the inputs of our deep learning MR analysis model were the enlarged ROIs.

Considering that the large amount of training data could improve the performance of the model, we used data augmentation method to increase number of ROIs including image flipping, image scaling, adding gaussian noise. In order to make all the ROIs have the same size, we resized them into 320 × 320, then the processed ROI were input into the conventional neural network (CNN). The detailed network architecture of the CNN is as following. The first part of the CNN includes one *64 7@7* convolution layer, a normalization module and a max pooling layer. After going through all these layers, we obtained a feature map of the input ROI roughly. The following structure of the CNN network contains six *64 3@3* convolution bottleneck modules and six *128 3@3* convolution bottleneck modules, in which all the bottleneck modules are employed from Resnet. The raw feature map of the ROI obtained at the first part was processed by these bottleneck modules sequentially, thus the feature of the ROI at different scales could be learned accordingly. The motivation behind this design of the network is that different scales of convolution bottleneck can help the CNN network learn feature with diverse scales. Features learned at shallow layers pay much attention to the detailed structure information while features learned at deep layers care more about the global information. Considering the target to analyze MR image is to predict the MVI level which is a global characteristic of an image, we directly use the output feature of the last *128 3@3* convolution bottleneck module which is a 512-dimensional feature vector and feed into the next fully connected layer (FC).

#### Step 2: feature fusion from multiple MR sequences

For each patient in 2015 HCC cohort, 3 continuous slices showing the maximal diameter of the tumor were first exported from six pulse sequences (i.e.,T1, T1A, T1V, T1D, T2, DWI), and then analyzed in our model. As mentioned above, the feature of an image from each sequence is extracted beforehand. Theoretically, we could use a CNN network with six branches sharing same weights to extract six modality images simultaneously, and then concatenated the learned six features as a fused one to feed into the FC layer. After passing the FC layer and the SoftMax, a confidence score identifying the level of MVI of the patient was obtained. However, the prediction performance of the CNN network by combining all six MR sequences was not as well as expected, and three of six sequences (i.e.T1, T1V, T1D) after empirically evaluation provides the best performance. More details could be referred to the experiment section.

#### Step 3: feature normalization

After concatenating the features from different pulse sequences, the fused feature was fed into a FC layer combined with a SoftMax classifier, which helped to normalize the feature into n×1 bin vector. Here *n* is the number of MVI levels.

#### Step 4: network training and testing

Considering that we had 3 continuous slices for each pulse sequences, anyone of the three slices could be used to characterize the MVI information from this sequence. Thus, for each patient, 27 different slice combinations from three MR sequences (i.e., T1, T1V, T1D) could be used as the inputs of the CNN network. In other words, for each patient, 27 training slices sharing the same label are given. Different from any data augmentation techniques, data shuffling like above is a unique way employed in our model thus the performance of our network could be further improved.

Given a testing sample, in the inference step, any one of the 27 different slice combinations could be used as the input of the trained CNN network. Without loss of the generality, we simply used the first slice of 3 continuous slices for each sequence.

### Histopathology

All surgical specimens were examined by 2 experienced pathologists, particularly to detect the presence of MVI. MVI was defined as the presence of tumor invasion in smaller intrahepatic vessels including a portal vein, hepatic vein, or a large capsular vessel of the surrounding hepatic tissue lined by endothelium that was visible only on microscopy [5]. MVI grade is classified as M1: the number of MVI < 5 and the distance of MVI ≤ 1 cm away from the tumor tissues, and M2: the number of MVI > 5 or the distance of MVI > 1 cm away from the tumor tissues, according to the practice guidelines for the Pathological Diagnosis of Primary Liver Cancer of China [47]. The histologic parameters ordinarily included Edmondson-Steiner grade, size, surgical margin, and MVI status of the tumor.

### Statistical analysis

Statistical analysis was performed using SPSS v.25 (IBM Inc., Armonk, NY, USA) and R software (R software version 3.5.2, R Project for Statistical Computing, http://www.r-project.org). The discrimination performance of the DL predictive model was measured by the area under the ROC curve (AUC) value in the primary training/validation set. Calibration curves were plotted to analyze the diagnostic performance of the predictive model in the overall cohort. Decision curve analysis was conducted to determine the clinical usefulness and net benefits of the developed predictive model.

Patients were consistently followed up since the date of surgical resection at intervals of 2 to 3 months. Recurrence-free survival (RFS) and overall survival (OS) were defined as the interval between surgery and detection of first recurrence or death. Patients were censored in case of emigration, or on 31 December 2020, whichever came first. Survival curves were plotted using the Kaplan-Meier method and compared by log-rank test. A two-tailed *p* value < 0.05 was considered statistically significant.

## Results

### Baseline clinical characteristics

Among the 321 patients enrolled in our study, histologic MVI was diagnosed in explanted tissue of 185 patients (57.6 %). Patients with MVI had higher ALT, AST, GGT and AFP levels than those without MVI. Patients with MVI and patients without were similar in their distribution of sex, hepatic virus infection, cirrhosis, Child-Pugh stage, TB, CB, ALB, and PT. Risk coefficient estimated by univariate analysis is summarized in Table 1.

**Table 1:** The clinical and histologic characteristics of primary cohort

Variable	No. of patients (*n* = 321)	Absent (*n* = 136)	Present (*n* = 185)	OR (95% CI)	*p* value
Age					
0, ≤ 50 years	91	33	58	1	
1, > 50 years	230	103	127	0.702 (0.425–1.157)	0.164
Sex					
0, Male	273	115	158	1	
1, Female	48	21	27	0.936 (0.504–1.738)	0.834
AFP					
0, ≤ 20 ng/mL	144	84	60	1	
1, > 20 ng/mL	177	52	125	3.365(2.118–5.347)	**< 0.001**
Ascites					
0, absent	301	130	171	1	
1, present	20	6	14	1.774 (0.664–4.741)	0.248
Hepatic virus infection					
0, absent	77	33	44	1	
1, present (HBV/HCV)	244	103	141	1.027 (0.612–1.723)	0.921
Cirrhosis					
0, absent	84	41	43	1	
1, present	237	95	142	1.425 (0.864–2.351)	0.164
ALT					
0, ≤ 40 U/L	229	108	121	1	
1, > 40 U/L	92	28	64	2.040 (1.220–3.412)	**0.006**
AST					
0, ≤ 40 U/L	226	111	115	1	
1, > 40 U/L	95	25	70	2.703 (1.597–4.573)	**< 0.001**
GGT					
0, ≤ 50 U/L	151	73	78	1	
1, > 50 U/L	170	63	107	1.590 (1.018–2.482)	**0.041**
TB					
0, ≤ 19 μmol/L	274	116	158	1	
1, > 19 μmol/L	47	20	27	0.991 (0.530–1.853)	0.978
CB					
0, ≤ 6.8 μmol/L	244	107	137	1	
1, > 6.8 μmol/L	77	29	48	1.293 (0.764–2.187)	0.338
ALB					
0, ≤ 40 g/L	91	40	51	1	
1, > 40 g/L	230	96	134	1.095 (0.671–1.787)	0.717
PLT					
0, ≤ 100*10^9^/L	40	19	21	1	
1, > 100*10^9^/L	281	117	164	1.268 (0.653–2.464)	0.483
INR					
0, ≤ 1.0	180	84	96	1	
1, > 1.0	141	52	89	1.498 (0.955–2.349)	0.078
PT					
0, ≤ 12 s	241	105	136	1	
1, > 12 s	80	31	49	1.220 (0.728–2.046)	0.45
Surgical size (cm)	321	4 (3–6)	6.3 (4.5–9.25)		**< 0.001**
Tumor encapsulation					
0, incomplete	194	88	106	1	
1, complete	127	48	79	1.366 (0.865–2.157)	0.18
Edmondson–Steiner grade					
I–II	141	77	64	1	
III–IV	180	59	121	2.467 (1.566–3.888)	**< 0.001**

*AFP* a-fetoprotein, *ALT* alanine aminotransferase, *AST* aspartate aminotransferase, *GGT* γ-glutamyl transpeptidase, *TB* total bilirubin, *CB* conjugated bilirubin, *ALB* albumin, *PLT* platelet count, *PT* prothrombin time, *INR* international normalized ratio, *OR* odds ratio, *CI* confidence interval

### Deep learning analysis of MR images

#### Effectiveness of single MR sequence

To validate the effectiveness of the single modality, we used the same model structure to extract features, but input classifier without feature fusion. We found that compared to the best result by modal combination among T1, T1D, and T1V, single modality didn’t perform well. We got 63.19% accuracy for T1V modal, 58.91% accuracy for T1D model and 66.66% for T1 modal.

#### Effectiveness of multi-sequences

Since we had 6 pulse sequences of MRI image data, we’ve done the ablation study to figure out which kind of modality combination could lead to the best classification result. As shown in Table 2, the combination of T1, T1D and T1V resulted in the highest accuracy (92.11%). Meanwhile, we noticed that the modality of DWI was not a proper modal for MVI classification.

**Table 2:** MVI classification accuracy comparisons for different combinations of modalities in 2015 HCC cohort

Combination	Accuracy
T1 T1D T1V	92.11%
T1 T1A T2	66.67%
T1 T1D T2	84.21%
DWI T1 T2	74.56%
DWI T1A T2	77.19%
T1V T1D	85.96%
T1V T1D T1	87.72%
T1V T1D T2	81.58%

*T1* T1-weighted imaging, *DWI* diffusion-weighted imaging, *T2* T2-weighted imaging, *T1A* T1-weighted imaging at arterial phase, *T1V* T1-weighted imaging at portal venous phase, *T1D* T1-weighted imaging at delayed phase

### Generalization between different cohorts

We then tested the model on external validation dataset collected in 2018. The result showed our model did not work well on 2018 dataset. We obtained the accuracy of 68.69%, the precision of 76.92%, the recall of 75.76% and the F1-score of 76.34%.

**Fig. 2:**
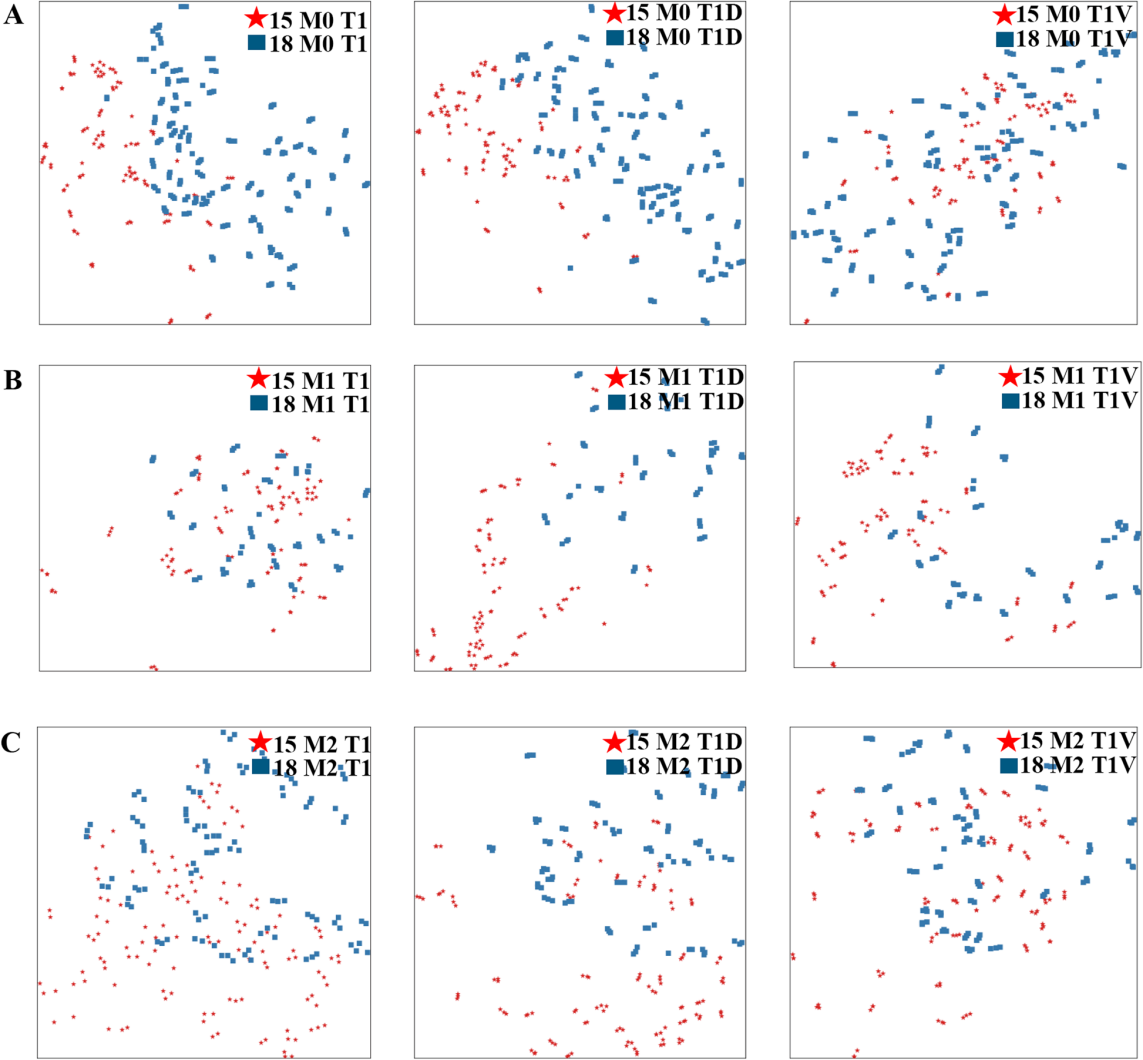
The distribution difference between the 2015 HCC cohort and the 2018 HCC cohort data from different modalities. **A** The distribution difference for M0 group with modalities T1, T1V and T1D, respectively. The 2015 HCC dataset and the 2018 HCC dataset can be obviously separated on the MR sequences of T1 and T1D, but are blended well on the sequence of T1V. **B** The distribution difference for M1 group with modalities T1, T1V and T1D, respectively. The 2015 HCC dataset and the 2018 HCC dataset can be obviously separated on the MR sequences of T1D and T1V, but are blended well on the sequence of T1. **C** The distribution difference for M1 group with modalities T1, T1V and T1D, respectively. The 2015 HCC dataset and the 2018 HCC dataset can be obviously separated on the MR sequences of T1D, but are blended well on the sequence of T1 and T1V. T1, T1-weighted imaging; T1V, T1-weighted imaging at portal venous phase; T1D, T1-weighted imaging at delayed phase

In order to analyze the reasons for the performance drop when the network was trained on HCC 2015 cohort and tested on 2018 HCC cohort data, we used t-SNE algorithm to reduce the dimensionality of the data into 2D such that we could display and analyze the difference between the two cohort data sets visually as shown in Fig. 2. The first row of Fig. 2 showed the distribution difference for M0 group with modalities T1, T1V, and T1D, respectively (Fig. 2A). From the first row of Fig. 2, we can see that the two datasets could be obviously separated on the sequences of T1 and T1D, but blended on the modality of T1V. Similar results were observed in the second and the third rows for the M1 and the M2 groups simultaneously (Fig. 2B, C). For M1 group, the 2015 HCC dataset and 2018 HCC dataset were separated on the MR sequences of T1D and T1V, but were blended well on the sequence of T1. Meanwhile, the 2015 HCC dataset and the 2018 HCC dataset were separated on the MR sequences of T1D, while blended on the sequences of T1 and T1V for M2 group. In other words, no matter how to select the combinations of the sequences among T1, T1D, T1V, at least one MR sequence cannot achieve satisfied classification results among M0, M1, and M2. The feature distribution inconsistency led to the poor classification performance of our model on 2018 HCC cohort data. We speculated that there were several reasons for this. The MRI imaging protocols changed slightly over the years from 2015 to 2018. In addition, the differences in MRI scanners, image parameters, and scanning technique of the users, could all account for the different performance of our model on the two cohorts. Finally, we used the deep learning model constructed above to predict MVI status of the overall cohort and the results were denoted as DL-predicted MVI status (DL-MVI).

### Predictors of survival

**Fig. 3:**
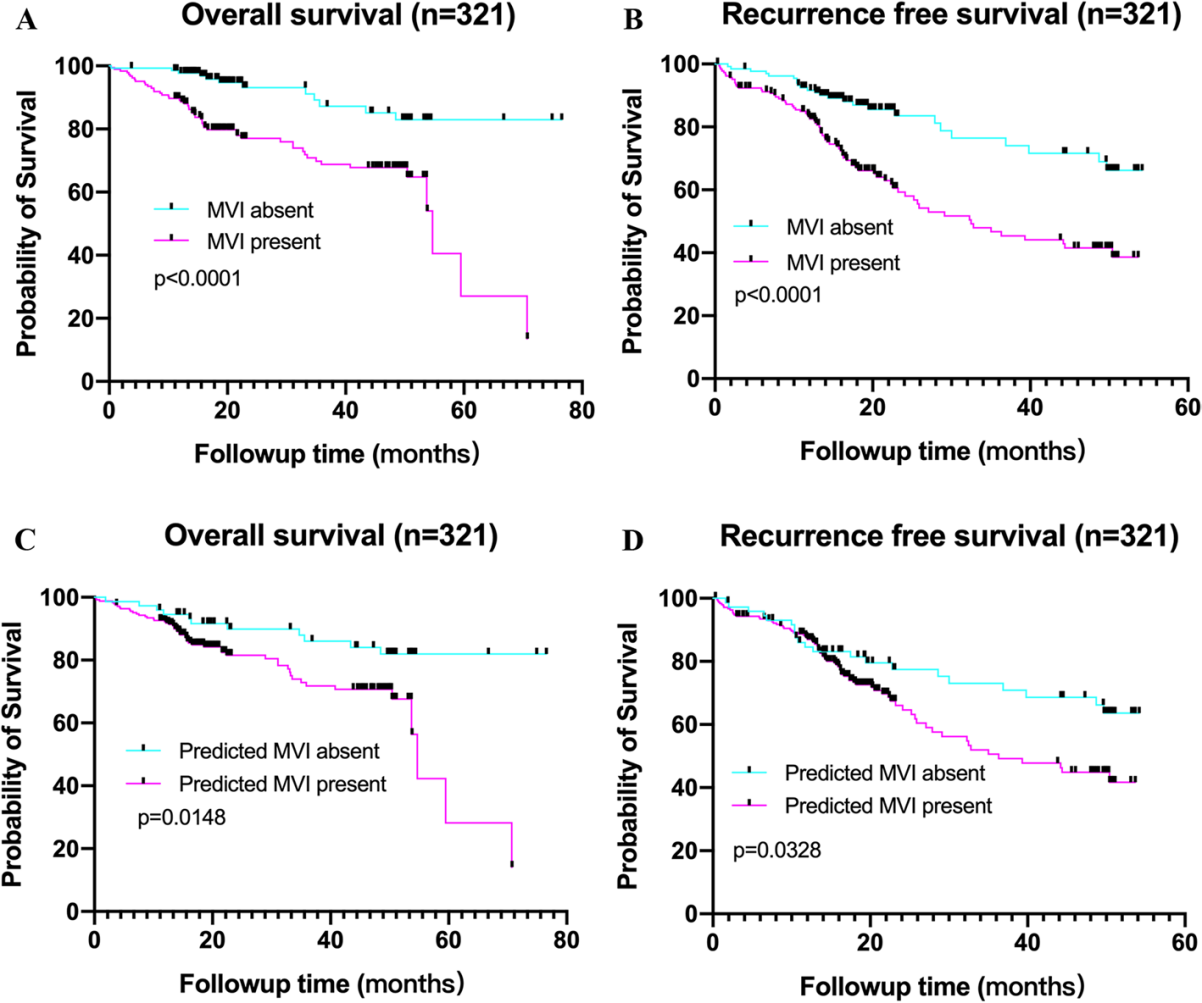
Survival curves according to histological and DL-predicted MVI status. **A**, **C** OS and **B**, **D** RFS curves scaled by pathologic MVI status and DL-predicted MVI status with Kaplan-Meier analysis. MVI, microvascular invasion; DL, deep learning; OS, overall survival; RFS, recurrence-free survival

As of Dec 2020, all the patients had completed the OS follow-up and RFS follow-up. The overall recurrence rate was 31.5% (101/321) and the overall death rate was 19.9% (64/321). The median OS of the patients was 59.5 months and patients with MVI had a median OS of 54.7 months (Fig. 3A). The median OS was 54.7 months for those with DL-predicted MVI presence and was not reached for those with DL-predicted MVI absence (Fig. 3C). The median PFS of the patients was 50.4 months, particularly 32.5 for patients with MVI and it was not reached for those without MVI (Fig. 3B). The median PFS was 36.3 months for patients with DL-predicted MVI presence and not reached for those with DL-predicted MVI absence (Fig. 3D).

### Construction of DL-based predictive model for MVI

**Fig. 4:**
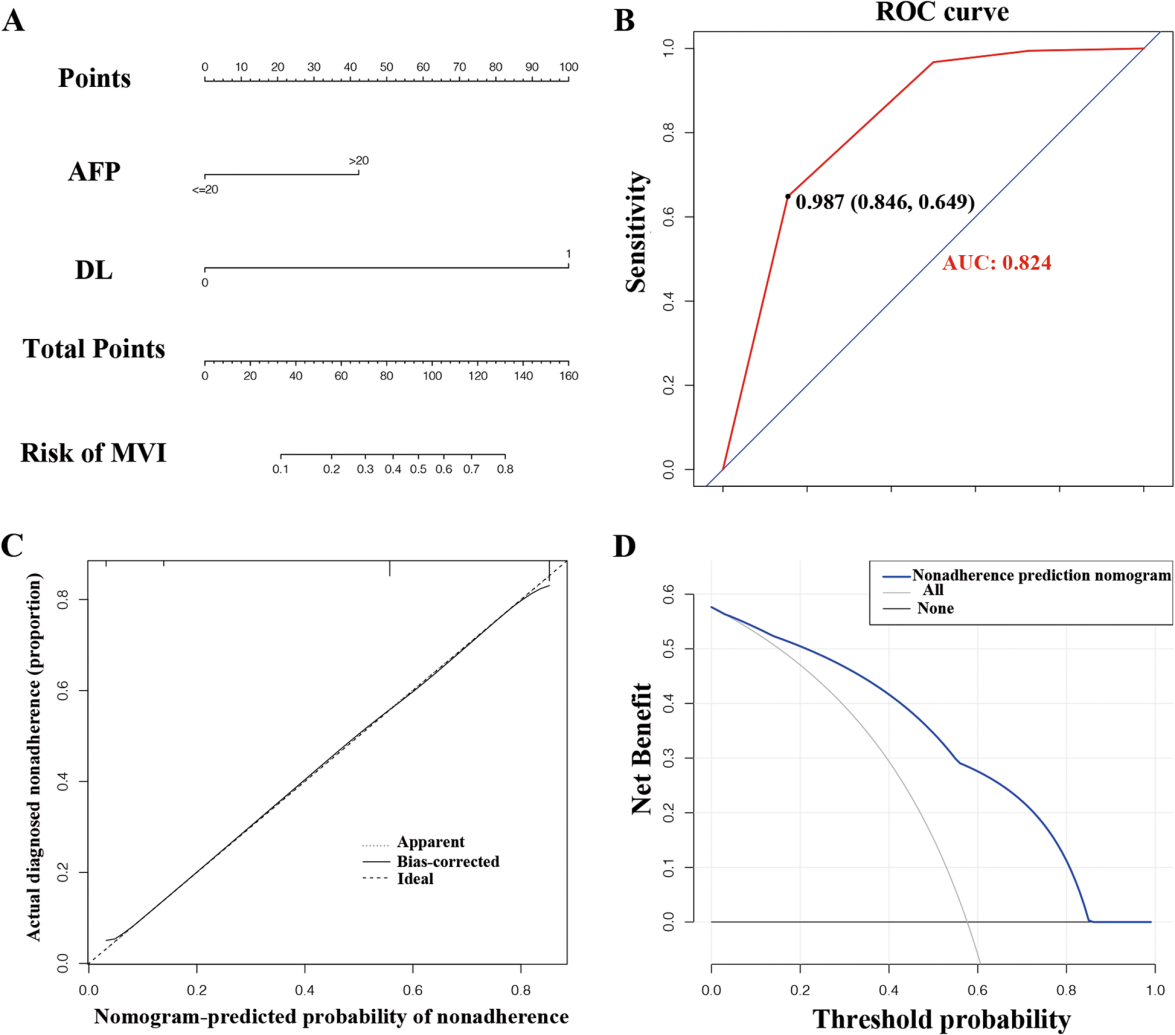
Nomogram for predicting microvascular invasion (MVI) probabilities, receiver operating characteristics (ROC) curves, calibration of the nomogram and decision curve in the overall patients. **A** A nomogram integrated DL-MVI and serum AFP level. **B** Receiver operating characteristic analysis of the nomogram. **C** Calibration curves of the nomogram in the overall datasets; X-axis is predicted probability of MVI. *Y*-axis is actual MVI. The diagonal dashed line indicates the ideal prediction by a perfect model. **D** Decision curve for the nomogram predicting the MVI in the overall patients

Among all clinical parameters, 4 clinical variables (ALT, AST, GGT, and AFP) were identified by univariate logistic analysis. In the multivariate regression model, only 2 predictors were independent prognostic factors of histologic MVI: higher AFP (> 20 ng/mL), DL-predicted MVI presence (Table 3). These independently associated risk factors were furthered enrolled to form the predictive model (Fig. 4A), described by the formula: *Y* = − 3.51 + 1.53 × AFP + 3.58 × DL-MVI. To apply the nomogram model clinically, there are mainly three steps. (1) Step 1: HCC patients with higher serum AFP level (> 20 ng/mL) get points of around 42.5 while those with low serum AFP level (≤ 20 ng/mL) get 0 points. (2) Step 2: for the DL-predicted MVI, the doctors can simply crop the tumor regions of three MR sequences (T1, T1D, T1V) as the inputs, then the DL model automatically has three outputs (predicted MVI absent, MVI-grade 1, and MVI-grade 2). MVI-grades 1 and 2 categories were together recorded as MVI present and get 100 points, while DL-predicted MVI absent get 0 point. (3) Step 3: sum the points gotten in step 1 and step 2. The total points correspond to the predicted probability of the nomogram model shown in Fig. 4A. The resulting DL-based predictive model demonstrated good accuracy in predicting the risk of MVI, with an AUC of 0.824 (Fig. 4B). The calibration curve of the model demonstrated good agreement between predicted and observed MVI in the primary cohort (Fig. 4C). The decision curve for the predictive model is demonstrated in Fig. 4D, the net benefit of the decision curve for the predictive nomogram is higher than that for assuming all patients have MVI when the threshold probability > 4%.

**Table 3:** Multivariate logistic regression analysis of factors associated with MVI

Variables	MVI	
	OR (95% CI)	*p* value
ALT (> 40 U/L versus ≤ 40 U/L)	1.617(0.763–3.427)	0.21
AST (> 40 U/L versus ≤ 40 U/L)	1.939(0.932–4.036)	0.077
GGT (> 50 U/L versus ≤ 50 U/L)	0.737(0.395–1.376)	0.338
AFP (> 20 ng/mL versus ≤ 20 ng/mL)	4.634(2.576–8.336)	**< 0.001**
DL-MVI (present versus absent)	35.738(14.027–91.056)	**< 0.001**

*ALT* alanine aminotransferase, *AST* aspartate aminotransferase, *GGT* γ-glutamyl transpeptidase, *AFP* a-fetoprotein, *OR* odds ratio, *CI* confidence interval, *DL-MVI* deep learning predicted microvascular invasion

## Discussion

The aim of our study was to investigate whether the DL-assisted model derived from large-scale clinical and imaging data, especially imaging features from DCE-MRI could be able to preoperatively predict MVI status and clinical outcomes in a cohort of 321 patients with HCC. Preoperative MVI status prediction is principle for clinicians to adopt appropriate therapeutic strategies, contributing to improve HCC patients’ overall survival. Histologic MVI has been claimed to be associated with advanced tumor stage [48] and poor HCC prognosis in many studies [15, 49]. Similar results were obtained in our primary cohort, patients with different DL-predicted MVI status andhistologic MVI had different clinical outcomes.

Recently, there have been several studies attempting to predict MVI using only clinical parameters. Radiomics has been recently viewed as a vital imaging technology in medical oncology [50]. Combing radiomics based on CT or MRI with clinical variables achieved the AUC from 0.796 to 0.906 [23, 51, 52]. However, the challenge of radiomics method is based on manually-defined precise boundary of the tumor, resulting in poor inter-reader reliability, and the results may not truly reflect the edge features of the target tumor.

The emerging DL method represents a new choice, due to its ability to integrate a large scale of clinical and imaging data. A recent study using DL based on preoperative CT showed a considerable efficacy (AUC 0.906) in predicting MVI [53]. Two other independent studies using DCE-MRI and 3D Convolutional Neural Networks instead of CT images to predict MVI achieved an AUC of 0.931 and 0.926 respectively [54, 55].

In our study, higher serum AFP level (> 20 ng/mL) and DL-predicted MVI presence were independently associated with histologic MVI by both univariate and multivariate logistic analysis, thus they were furthered included in the predictive model. The resulting DL-based predictive model demonstrated good accuracy in predicting the risk of MVI, with an AUC of 0.824. Besides AFP level, preoperative serum biomarkers like low platelet counts were reported to exert an unfavorable impact on the recurrence of patients with small HCC after liver resection [56], while it is not associated with histologic MVI in our study and thus not included in our model. Similarly, a previous study achieved an AUC of 0.81 by combining DL with 3D convolutional neural network for noninvasive prediction of MVI in HCC [57].

Algorithmically, the DL model applied in this study is a multi-input network. As we used six image sequences for MVI prediction, we first evaluated the effectiveness of single modality and then multi-modalities. It turned out that single modality did not perform well as compared to multi-modalities. Furthermore, the combination of T1, T1D, and T1V resulted in the highest accuracy (92.11%) in the training cohort. However, this model did not perform well on the validation dataset. In order to analyze the reasons for this apparent discrepancy between the 2015 training cohort and the 2018 validation cohort, we used t-SNE algorithm to reduce the dimensionality of the data into 2D such that we could display and analyze the difference between the two cohort data sets visually. The results suggested that feature distribution inconsistency led to the bad classification performance of our model on 2018 HCC cohort data.

Several limitations of this study should be noted. First, because of the inherent character of a retrospective study, potential selection bias is possible. Only patients meeting inclusion criteria were selected, while many patients who were clinically considered as  “high-risk” for MVI but did not undergo surgical resection were excluded. The potential selection bias may impair the application of our model in an expanded population of HCC patients. Besides, the DCE-MRI sequences were acquired from different scanners. Although standardized processing was performed to reduce the impact of differences in scanners and image parameters, some potential differences, such as scanning technique, could still exist. Also, this study was a single-center experience limited to our medical center, and the study results should be validated and reproduced by external medical centers.

## Conclusions

In conclusion, we systematically investigated the large-scale clinical and MR imaging data of patients with HCC undergoing surgical resection with the assistance of DL for noninvasive prediction of MVI. Our predictive model integrating deep learning and serum AFP level demonstrated good performance for predicting MVI and clinical outcomes in patients with HCC.

## Supplementary Information

**Additional file 1: Supplemental Table 1.** Imaging parameters.

## Data Availability

All data generated during this study are listed in this article. The data are available from the corresponding author upon reasonable request.

## Abbreviations

MVI: Microvascular invasion
HCC: Hepatocellular carcinoma
DCE-MRI: Dynamic contrast-enhanced magnetic resonance imaging
DL: Deep learning
CNN: Conventional neural network
AUC: Area under curve
OS: Overall survival
RFS: Recurrence-free survival
CI: Confidence interval
OR: Odds ratio
