# Supplementary material for: Deep-learning-based analysis of preoperative MRI predicts microvascular invasion and outcome in hepatocellular carcinoma
Source: World J Surg Oncol. 2022 Jun 8;20:189. doi: 10.1186/s12957-022-02645-8 (PMC9178852; doi:10.1186/s12957-022-02645-8)
Supplement: Supplementary file 1 — Additional file 1: Supplemental Table 1. Imaging parameters. [file 12957_2022_2645_MOESM1_ESM.docx]

Supplemental Table 1. Imaging parameters

| Parameters | TR  (msec) | TE  (msec) | BW  (Hz/pixel) | FOV  (mm2) | Matrix | Section  thicknenss (mm) | Acquisition time (sec) |
| --- | --- | --- | --- | --- | --- | --- | --- |
| Respiratory-triggered  T2-weighted imaging | 4918 | 106 | 195 | 285×380 | 384×273 | 5.5 | respiration-  dependent |
| Free-breathing DWI | 5100 | 55 | 1565 | 285×380 | 192×154 | 5.5 | 74 |
| Breath-hold T1-  weighted VIBE imaging | 3.47 | 1.36 | 400 | 308×380 | 320×240 | 3 | 15 |

TR, Repetition time; TE, Echo time; BW, Bandwidth; FOV, Field of view; DWI, Diffusion-weighted Imaging; VIBE, Volumetric interpolated breath-hold examination.
